# Effects of Microstructure Modification by Friction Surfacing on Wear Behavior of Al Alloys with Different Si Contents

**DOI:** 10.3390/ma15051641

**Published:** 2022-02-22

**Authors:** Malte R. Schütte, Jonas Ehrich, Dominic Linsler, Stefanie Hanke

**Affiliations:** 1 MicroTribology Center μTC, Fraunhofer Institute for Mechanics of Materials IWM, 76131 Karlsruhe, Germany; dominic.linsler@iwm.fraunhofer.de; 2 Materials Science and Engineering, Institute for Metal Technologies, University of Duisburg-Essen, 47057 Duisburg, Germany; jonas.ehrich@uni-due.de (J.E.); stefanie.hanke@uni-due.de (S.H.)

**Keywords:** friction surfacing, aluminum, silicon, microstructure modification, pin-on-disc, lubricated sliding wear, aluminum/steel tribocouples

## Abstract

In this work, Al alloys with 6.6%, 10.4%, and 14.6% Si were deposited as thick coatings by Friction Surfacing (FS), resulting in grain refinement and spheroidization of needle-shaped eutectic Si phase. Lubricated sliding wear tests were performed on a pin-on-disc tribometer using Al-Si alloys in as-cast and FS processed states as pins and 42CrMo4 steel discs. The chemical composition of the worn surfaces was analyzed by X-ray photoelectron spectroscopy (XPS). The wear mechanisms were studied by scanning electron microscopy (SEM) and focused ion beam (FIB), and the wear was evaluated by measuring the weight loss of the samples. For the hypoeutectic alloys, spheroidization of the Si phase particles in particular leads to a significant improvement in wear resistance. The needle-shaped Si phase in as-cast state fractures during the wear test and small fragments easily detach from the surface. The spherical Si phase particles in the FS state also break away from the surface, but to a smaller extent. No reduction in wear due to FS was observed for the hypereutectic alloy. Here, large bulky primary Si phase particles are already present in the as-cast state and do not change significantly during FS, providing high wear resistance in both material states. This study highlights the mechanisms and limitations of improved wear resistance of Si-rich Al alloys deposited as thick coatings by Friction Surfacing.

## 1. Introduction

**Figure 1:**
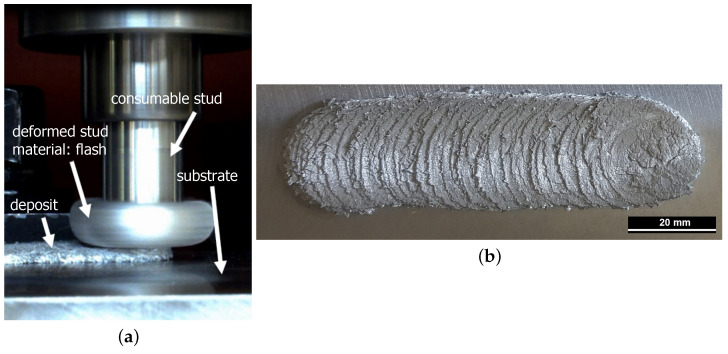
(**a**) Photograph of the FS process and (**b**) photograph of an FS deposited coating from an Al alloy with 14.6% Si.

Friction Surfacing (FS) is generally used to apply metal-based coatings in a solid state, i.e., without bulk melting the material being deposited. For this reason, it is of particular interest for materials that are considered difficult or even impossible to weld [1,2,3]. The repair or reconditioning of worn or damaged components was identified early on as a promising application for FS [4,5]. Further technically useful features of the process include the possibility to deposit dissimilar materials without dilution and to apply several layers of different materials on top of each other or overlapping to create functional coatings, transition layers with a gradient in a specific property, or an approach of additive layer manufacturing [6,7,8]. Adding a powder into the stud allows the formation of composites during deposition, although the resulting particle distribution is inhomogeneous due to the complex and insufficiently understood material flow [9,10,11,12]. A photograph of the ongoing process is shown in Figure 1a. The process starts with a stud of the coating material. Its face is pressed with a defined axial force onto the substrate to be coated, while the stud rotates around its longitudinal axis. Frictional heating occurs in the contact, and the temperature of the material increases until the stud face adheres to the substrate. Now, the rotational motion of the stud no longer occurs at the interface with the stationary substrate but, rather, by plastic flow within a shear plane in the thermally softened material in the stud tip. This soft, plastically deforming stud material is often referred to as a “quasi-liquid layer” or shear zone [5,13,14]. Now, a transverse motion is superimposed by moving the machine table on which the substrate is clamped. Along the path, some of the softened material is deposited from the stud tip onto the substrate, leaving a coating with a width similar to the stud diameter (Figure 1b). Along the outer edge of the stud, the softened material cannot fully transfer the axial forging force onto the substrate, resulting in a lack of bonding along a narrow area at the edges of each coating layer. In addition, some of the soft material at the edge of the stud forced out of the contact zone by the axial force and forced up around the stud, creating a flash [4,13]. Previous studies have shown that the bond strength of the metallurgical bond in FS composites depends on the process parameters, material pairing, and surface properties of the substrate [15,16]. The pin specimens used in this study, which were fabricated by the FS process, show strong and homogeneous bonding, which is not investigated further here. Shear deformation at elevated temperature, which is essential for the FS process, leads to thermomechanical processing of the deposited material. Dynamic recrystallization occurs, often resulting in fine, equiaxed grains in the coatings [8,17]. Phase dissolution at process temperatures is favored by the applied mechanical energy, and subsequent cooling rates are usually very high. This results in coatings containing supersaturated solid solution phases, non-equilibrium precipitates, or martensite [2,18,19]. Mechanical processing, additionally, also causes non-soluble phases to break up or become spherical [3,20]. Such microstructural changes have been shown to be beneficial to the wear resistance of a material and result in less wear in FS-processed material compared to a conventional equilibrium microstructure [9,11,21]. Silicon (Si) is an important alloying element of aluminum (Al). Si favors castability in Al alloys, which is why Al-Si alloys are often used as cast alloys for engine blocks or pistons [22]. Silicon also improves corrosion and wear resistance [22,23]. Magnesium (Mg) is added to Al-Si alloys to increase strength by increasing precipitate formation. Due to its high affinity for oxygen (O), higher Mg contents can result in increased oxidation of products, which can lead to material embrittlement as part of precipitation coarsening [22]. Copper (Cu) in Al alloys promotes further formation of coherent precipitates that increase yield strength. The trace element iron (Fe), among others, promotes the formation of poorly soluble intermetallic phases consisting of Al and Si. These effects are compensated by the addition of manganese (Mn). Small amounts of other elements in Al alloys, e.g., chromium (Cr) and zinc (Zn), promote the formation of complex intermetallic hard phases resulting in a more brittle material [22,23]. In the present study, three Al-base alloys with different Si contents are investigated. Due to the comparable amounts of alloying elements—apart from Si—, it is assumed in this study that the influence of the intermetallic phases is similar in all alloys. Al cast alloys with 6% to 25% Si are used commercially in vehicle construction, including hypoeutectic casting alloys, which are used for, e.g., cylinder heads (approximately 10% Si) or chassis components (approximately 5% to 7%). Hypereutectic alloys are used, for example, for engine parts or pistons (13% to 25% Si). In order to exclude influences of other alloying elements on both the FS processing characteristics and on the wear behavior of the materials, customized Al-Si alloys were produced in which only the Si content was adjusted following commercially available, applicable alloys [24]. The microstructure of hypoeutectic Al-Si alloys (in this study: 6.6% Si and 10.4% Si) in the as-cast condition consists of coarse Si needles, which have a negative effect on the mechanical properties. Under loading conditions, local stress peaks occur along the Si needles, which can lead to cracking and, thus, reduce strength. With regard to the causes of wear in Al-Si cast materials, the size of the dendrites and the quantity, size, morphology, aspect ratio, and distribution of the hard Si phases can play a significant role [25]. Spheroidizing of the Si particles in Al alloys is possible by adding small amounts of strontium (Sr), which favors the rounding and reforming of the Si particles, improving the isotropy of properties and wear resistance [23,25,26]. Friction Surfacing of cast Aluminum with high Si content as coating material has not been reported in the literature yet. The thermomechanical processing of Al-alloys with high Si content by FS is of great interest from an engineering point of view, as it is expected to result in a modified microstructure with advantages for wear, corrosion, and fatigue loads. The application of FS to cast Al-based alloys with high Si content has not yet been studied in detail. The feasibility and suitable processing parameters were presented by the authors in a previous study [20]. It can be expected that the Si phase, which is not soluble in the Al-base solid solution, will be modified in its morphology and redistributed. Cast Al-based alloys are used for applications with tribological loads, where the hard Si phases provide the required wear resistance for the soft Al matrix [27,28,29,30,31]. Severe plastic deformation in solid-state joining processes, such as friction stir processing, has successfully demonstrated particle refinement in various aluminum alloys. In addition, the fine, homogeneously distributed secondary particles in Al-Si-alloys lead to an enhancement of the mechanical properties [32]. The microstructural refinement during SPD improves the tribological properties. It has been shown that wear resistance is improved by breaking up and evenly distributing the Si particles inside the Al-matrix [32]. For the related solid-state process Friction Stir Processing, in which a non-consumable tool is used to thermomechanical process the near surface material of a component, an improvement in the wear resistance of a cast Al-based alloy has been shown by Singh et al. [33]. In this study, FS is used, firstly, for spheroidize the Si particles and, secondly, to recrystallize the microstructure, so that, besides refinement and rounding of the Si particles, there is also massive grain refinement by plastic deformation, which is expected to lead to an enhancement of the mechanical properties. Applying layers from high-Si Al-base alloys by FS will make their wear resistance available locally also on components from wrought Al alloys. The main aim of this work is to clarify the feasibility and the potential of improving the wear resistance of Al-alloys by deposition high-Si Al-alloys by FS.

## 2. Materials and Methods

### 2.1. Materials and Sample Manufacturing

Three aluminium alloys were custom-made. For this purpose, bar-shaped base material was melted from a single production batch of alloy AA6060, and Si was added up to different contents. The Si-rich alloys were cast into cylindrical molds. The castings were then machined into studs with a diameter of 20 mm and a length of 120 mm, which were finally used as consumable studs for applying the FS layer to the substrate. Using optical emission spectroscopy, the chemical compositions of the three casting alloys were determined and are listed in Table 1.

**Figure 2:**
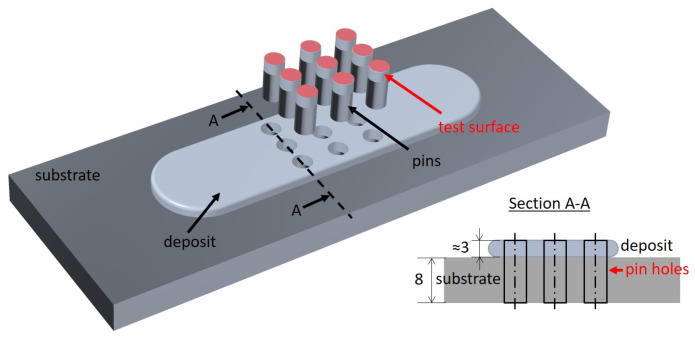
Illustration of pin sample extraction.

The coatings were deposited using a custom-designed friction surfacing machine from H. Loitz Robotik (Hamburg, Germany) at the department Solid State Materials Processing within the Institute of Materials Mechanics at Helmholtz Center Geesthacht, Germany. Details of the FS processing and resulting coating properties have been published in Reference [20]. Cast studs of the three alloys were deposited onto a substrate of AA2024 aluminum alloy. During the wear tests, the substrate material is not in contact with the counter body and is, thus, not considered further in this study. The pin samples for wear tests were extracted from homogeneously deposited coatings with a thickness of approximately 3 mm (see Figure 1). The substrate below the coating is partially included in the pins to provide sufficient length for clamping. The procedure for extracting the pin samples is shown schematically in Figure 2.

For wear tests in a Pin-on-Disc tribometer, the pins (body) were manufactured to dimensions of a diameter of 5 mm and a length of approximately 4 mm. The test surfaces of the pins were ground in a horizontal surface grinder (G&N, Erlangen, Germany) using abrasive corundum 200 and then polished by metallographic methods with 9 μm and 1 μm diamond suspension. Surface roughness parameters were controlled in sample preparation: Core roughness $R_{k}$, reduced peak height $R_{pk}$, and reduced valley depth $R_{vk}$ all range below a maximum value of 0.2 μm. The total height of profile waviness $W_{t}$ is < 1 μm. The mean values and standard deviations of the hardness (HV0.2) of the pins can be seen in Table 2. The large standard deviations result from the inhomogeneous microstructure.

**Figure 3:**
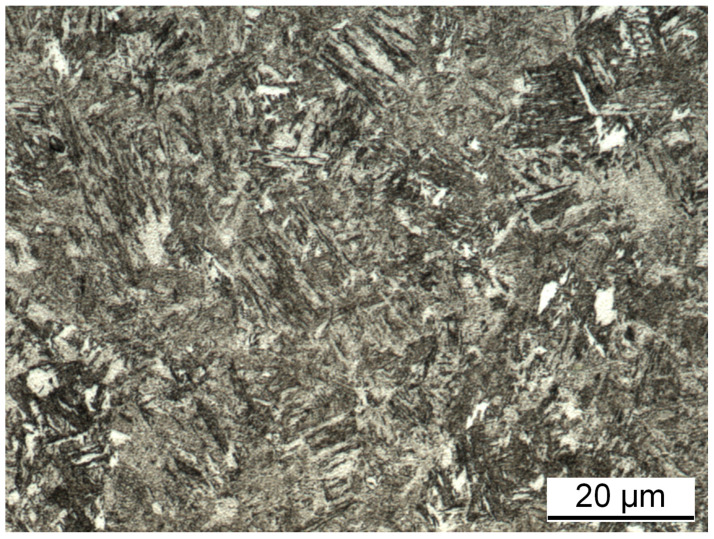
Microstructure of 42CrMo4 disc; approximately 90% bainite, 5% ferrite, and 5% martensite (etching: ethanol with 3% HNO${}_{3}$).

Discs with a diameter of 70% and a thickness of 5 mm made from steel 42CrMo4 (AISI 4140) are used as counter bodies. The average hardness of the counter body discs was measured at 312.6(20) HV10. The microstructure is shown in Figure 3 and contains approximately 90% bainite, 5% ferrite, and 5% martensite. For final processing, the discs were polished using barrel finishing. Topography was measured using white light interferometry (Contour GTK, Bruker, Billicera, USA). The resulting surface reveals roughness values of R${}_{pk}$ = (0.25±0.04)μm, R${}_{k}$ = (0.58±0.269)μm, and R${}_{vk}$ = (0.2±0.09)μm and a waviness of W${}_{t}$ = (1.71±0.62)μm. The chemical composition of the discs (according to material standard) is listed in Table 3.

### 2.2. Metallography and Microscopy

For the analysis of the initial pin microstructure, samples were cut with the cutting machine Accutom-50 (Struers GmbH, Willich, Germany) from the deposited material (friction surfacing), as well as from the material in as-cast state. The cut-outs were embedded, ground, and polished by standard metallographic methods. A final polishing step with 0.05 μm colloidal silica suspension (Masterprep Polishing Suspension, Buehler, Düsseldorf, Germany) and vibratory polishing (Vibromet, Buehler, Düsseldorf, Germany) was necessary for optical light microscopy. For the color-etched microstructures, the samples were anodized in Barker reagent ( 5 mL HBF4 (48%) in 200 mL aqua dest.) at 22 ${}^{\circ }C$ for 120 s at a voltage of 20 V using the electrochemical etching device LectroPol-5 (Struers GmbH, Willich, Germany). The samples were analyzed by light microscopy under crossed polarized light and a sensitive tint filter (BX-10, Olympus, Duesseldorf, Germany).

### 2.3. SEM/FIB

Images of the surface, as well as of surface sections, were generated with Focused Ion Beam (FIB)/Scanning electron microscope (SEM) Dual Beam instrument Helios 650 (FEI Company, now Thermo Fisher, Waltham, MA, USA). Additionally, an SEM (LEO 1530 GEMINI (Carl Zeiss Microscopy GmbH, Munich, Germany)) was used.

### 2.4. XPS

Furthermore, chemical analysis of the surface was carried out using X-ray photoelectron spectroscopy (XPS; VersaProbe II, Physical electronics, Chanhassen, MN, USA). Before determining the chemical compositions of the surfaces, the analyzed pin surfaces were washed in acetone and isopropyl alcohol. Argon ion sputtering allows chemical depth profiling of the surfaces, here up to 1100 nm. The sputtered area measures 200 μm× 200 μm and was selected to measure the composition of a representative surface without chipping or the like.

### 2.5. Pin-on-Disc Tribometer

**Figure 4:**
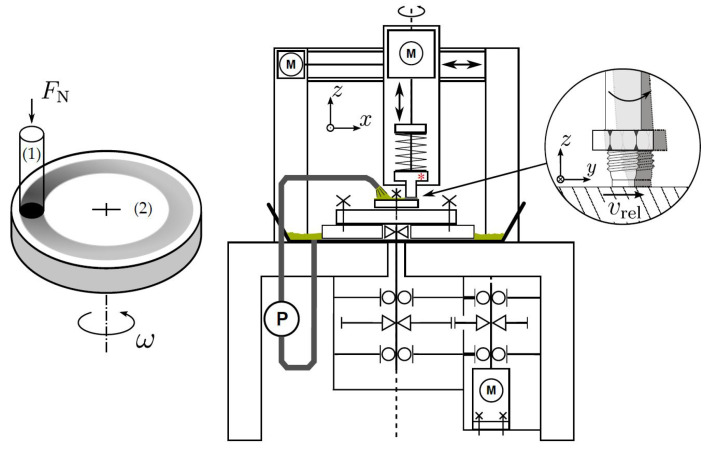
Illustration of the pin-on-disc tribometer taken from Schlarb [34].

The tribological tests were carried out on a pin-disc tribometer. A pin (body) is pressed eccentrically onto a rotating disc (counter body). The structure of the tribometer is shown in Figure 4. The radial run-out of the disc, i.e., the difference in height between the highest and lowest point of the disc in the mounted state, was set for the tests using a dial gauge to an accuracy of z < 2.5 μm. The pins had a diameter of 5 mm, and the radius of the track on the disc was 32 mm. The 5W30 Titan Longlife III engine oil (Fuchs Schmierstoffe GmbH, Mannheim, Germany) at 70 ${}^{\circ }C$ was used for lubrication. The tests consisted (at a friction velocity of 0.8 m $s^{-1}$) of a 90-min operating point at 8 MPa (or 5.3 MPa) and a subsequent operating point at 15.9 MPa (or 10.6 MPa) for 48 h and were performed at least three times for each material and state.

## 3. Results

### 3.1. Al-Alloys Microstructure

**Figure 5:**
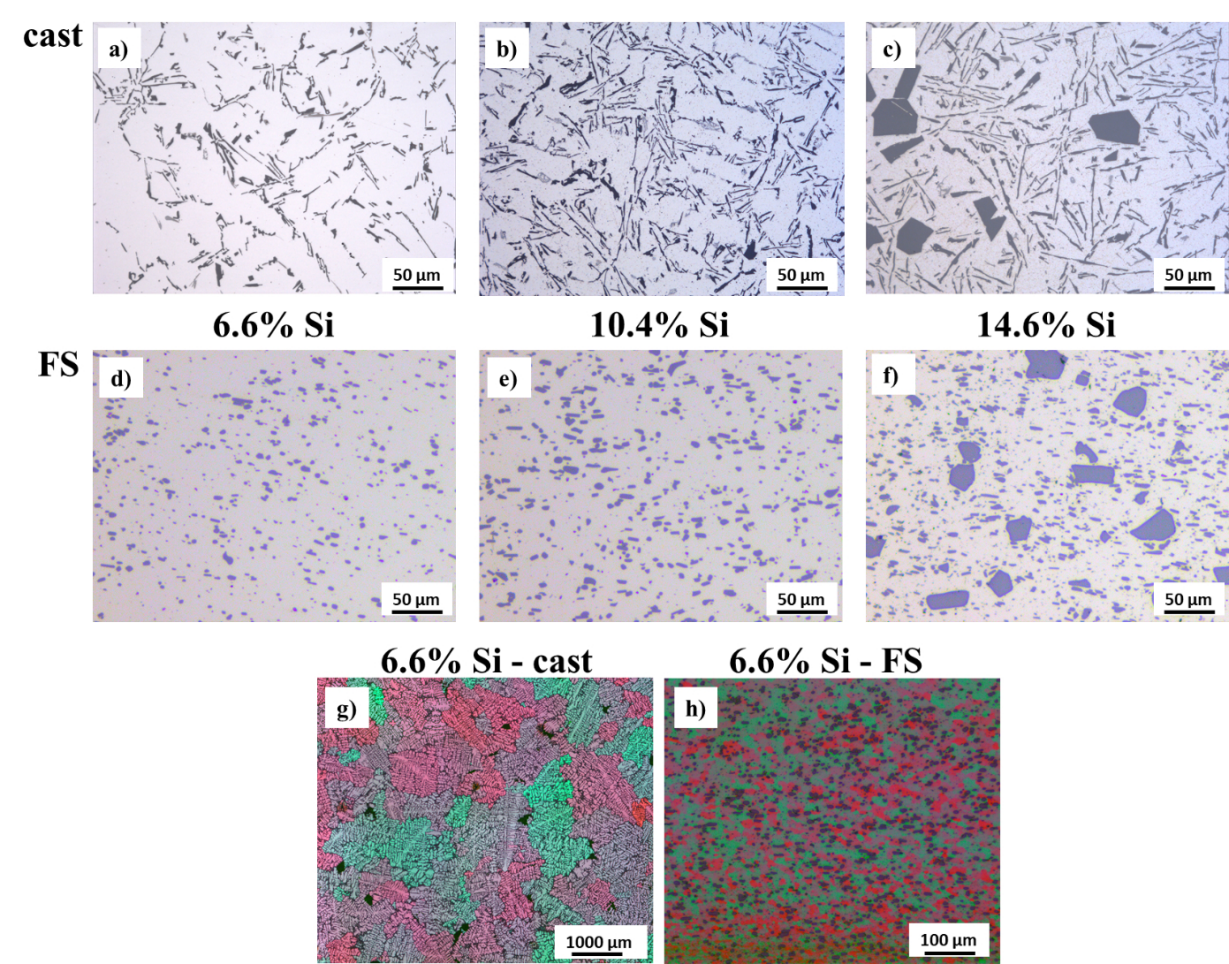
Light microscopic images showing the microstructure of the Al alloys. As-cast state: (**a**,**b**) hypoeutectic, Si needles; (**c**) hypereutectic, Si needles, Si primary phase particles. FS coatings: (**d**,**e**) hypoeutectic, fine dispersed Si particles; (**f**) hypereutectic, fine dispersed Si particles, Si primary phase particles. After electrolytical etching observed under polarized light 6.6% Si alloy: (**g**) as-cast-coarse grain size, (**h**) FS coating-fine-grained recrystallized microstructure.

The microstructure of the three processed Al alloys in as-cast state and after deposition as FS coatings is shown in Figure 5. In the as-cast state, Si needles dominate the microstructure in the two hypoeutectic alloys (Figure 5a,b), while the hypereutectic alloy additionally contains bulky primary Si phase particles (Figure 5c). After FS deposition, the Si needles have largely transformed into finely dispersed, almost spherical Si particles, while the bigger primary Si particles in the hypereutectic alloy remain almost unchanged (Figure 5d,e). The grain structure can be observed after etching using polarized light. The grains in the Al-alloy with 6.6% Si in as-cast state and after coating deposition are shown in Figure 5g,h. Grain refinement through dynamic recrystallization by a factor of approximately 20 is clearly observable. The average grain size for the 6.6% Si alloy in as-cast state is (440.8±117.52)μm and in FS state (20.8±2.1)μm (determined by linear intercepts method according to DIN EN ISO 643).

### 3.2. Friction Coefficients and Wear Rates

**Figure 6:**
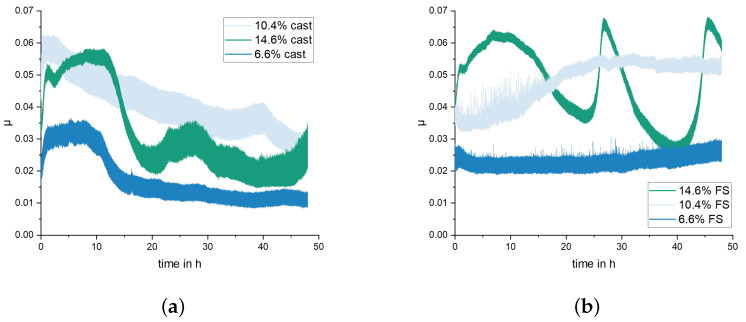
Coefficient of friction over test duration for (**a**) as-cast and (**b**) FS state.

Figure 6 shows the friction coefficient curve for one representative test for each material and state. It should be noted, however, that the other tests with 6.6% and 10.4% Si show very similar course, while, for the hypereutectic alloy, all experiments showed a variation in the coefficient of friction between μ = 0.03 and 0.07 over time, as shown in Figure 6b, with time differences in the range of 15 h to 20 h. The coefficient of friction with hypoeutectic alloys increases slightly for FS state and decreases slightly with cast alloy. The lowest coefficient of friction was achieved in both FS and cast state with the hypoeutectic alloy with 6.6% Si. Apart from the large scatter for the alloy with 14.6% Si, similar values of friction coefficients were obtained for 10.4% and 14.6% Si that were about twice as high as for 6.6% Si.

**Figure 7:**
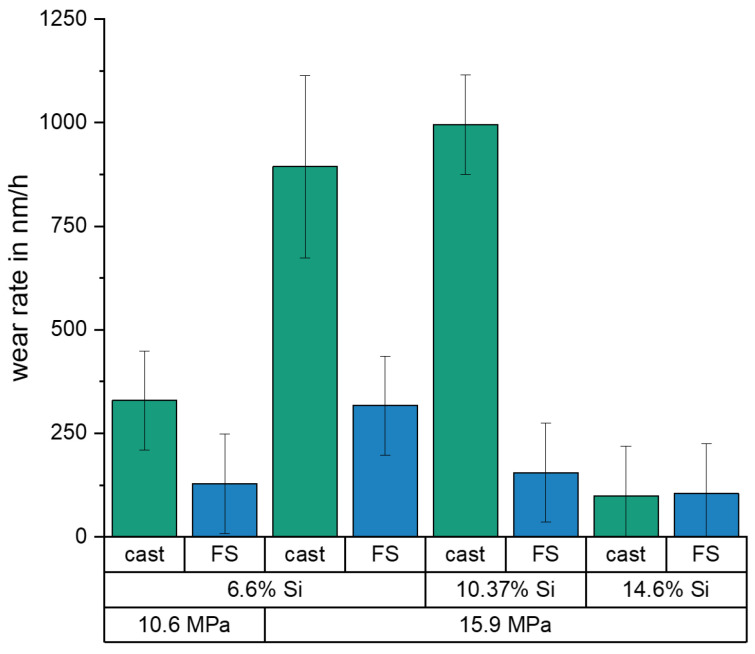
Wear rates (weight loss) at end of tests with different contact pressures (10.6 MPa and 15.9 MPa), for the three alloys (6.6%, 10.4% and 14.6% Si) in FS and as-cast state.

Differences in the wear rate of the pins can also be observed (Figure 7), which do not correlate with the trends in the coefficients of friction. The wear rate was calculated by dividing the weight loss by the experimental time. The weight loss was determined by weighing the samples with an analytical balance (Kern, ABJ320-4NM, uncertainty 0.3 mg) before and after the test. The alloy with 14.6% Si shows a small difference in wear rate between FS coating (106 nm $h^{-1}$) and as-cast state (99 nm $h^{-1}$) within the uncertainty of the measurement. However, the hypoeutectic alloys show a significant difference. The largest difference is observed for the alloy with 10.4% Si, for which the FS material displays lower wear rates (155 nm $h^{-1}$) by a factor of ≈6, compared to the as-cast state (996 nm $h^{-1}$). The material with 6.6% Si shows a comparable difference, as shown in Figure 7, which is more pronounced at the higher contact pressure of 15.9 MPa, but also clearly observable for the low contact pressure of 10.6 MPa. Wear on the steel discs was too low to be measured by the applied analytical balance.

### 3.3. Wear Mechanisms

**Figure 8:**
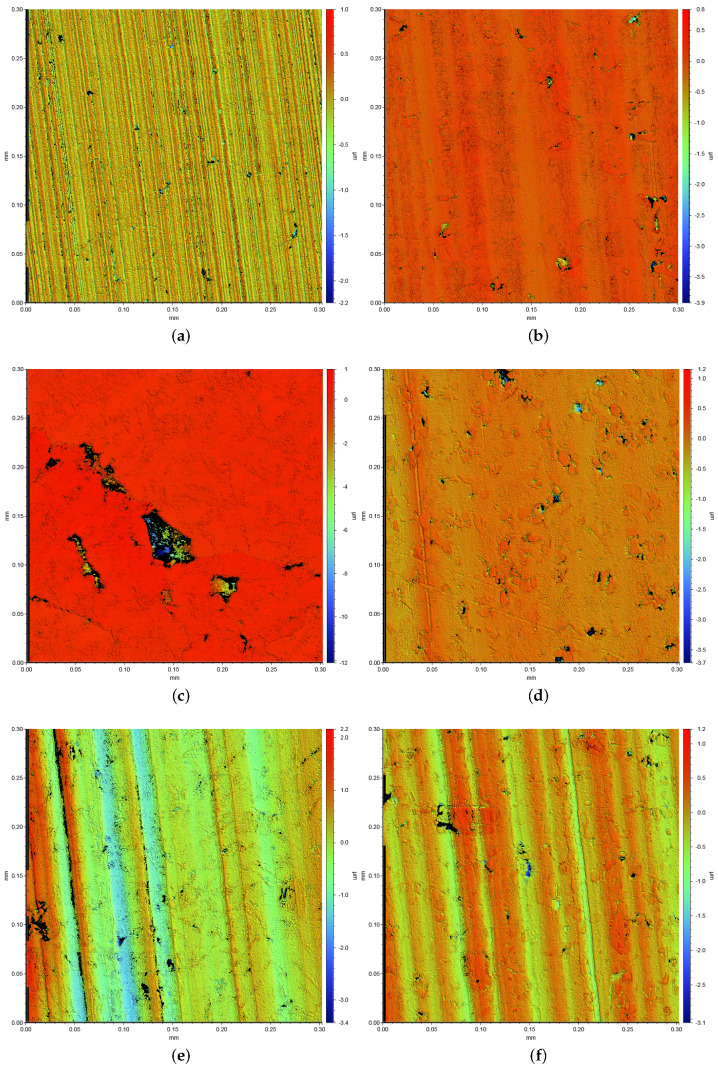
WLI images of the pin surfaces following the wear tests at 15.9 MPa MPa contact pressure. Note the differences in scaling of the height values. (**a**) 6.6% Si as-cast, (**b**) 6.6% Si FS, (**c**) 10.4% Si as-cast, (**d**) 10.4% Si FS, (**e**) 14.6% Si as-cast, and (**f**) 14.6% Si FS.

Figure 8 shows worn surfaces of the pins with three different Si contents in FS and as-cast state. It is noticeable that the wear mechanisms differ significantly depending on the alloy and microstructure. The surfaces with 14.6% Si show mainly abrasion by microploughing, independent of the material state. This leads to the formation of grooves with a depth of up to three microns and a width of (0.01±2.02)μm (FS) and 0.05 mm (cast) in the measured surface sections. On the other hand, the samples with 10.4% Si appear, generally, quite smooth with pits or breakouts of various sizes with a depth of up to 50 μm distributed over the surface. For the surfaces with 6.6% Si, a distinction must be made between the FS and as-cast state. Although both pins also display pits or breakouts, the cast pin also presents fine grooves, while the FS pin has a smooth surface similar to the pins of 10.4% Si; see Figure 8.

**Figure 9:**
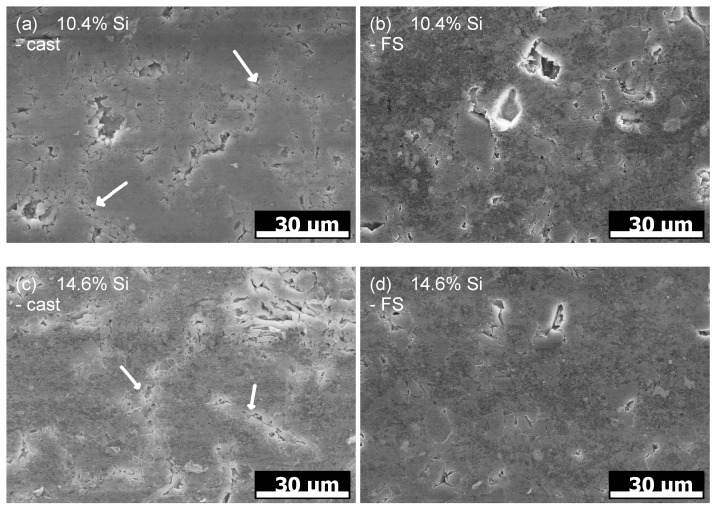
SEM images of pin surfaces after testing. (**a**) 10.4% Si as-cast and (**b**) FS state. (**c**) 14.6% Si as-cast and (**d**) FS state.

A closer look at the worn surfaces of pins with 10.4% Si and 14.6% Si is shown in Figure 9. The type of surface appearances observable are comparable for all four images. There are regions of dark, grainy appearance, and, in between, the Si phase is recognizable. It appears that the previously observed pits result from Si phase particles breaking out from the worn surface. For the as-cast state with both Si contents, it can be observed that the needle-shaped Si phase fractures (marked with arrows) and fragments detach from the surface. This leaves elongated regions of brighter appearance in the SEM images, which contain a high number of cracks and pits. In the FS state, Si phase particles also detach, leaving local pits, but, here, the spheroidized Si particles are distributed more uniformly in the material and show significantly fewer cracks.

**Figure 10:**
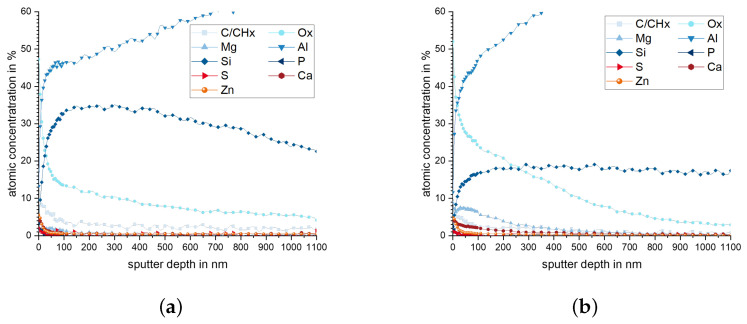
Chemical composition of elements with more than 2% atomic concentration (measured by XPS) of 10.4% Si pin surfaces after wear tests in (**a**) as-cast state and (**b**) FS state.

Chemical composition of the worn surfaces was determined using XPS. After the experiment, the elements Mg, Zn, Ca, P, and S could be determined in addition to Al and Si, which are the main elements of the tested alloys. Figure 10 shows the XPS results for pins with 10.4% Si in as-cast and FS state. The cast material (Figure 10a) reaches a Si content of up to 35% below the surface. However, the Si content decreases from a depth of 180 nm to 22% at a depth of 1 μm. In the case of the FS state (Figure 10b), the Si content ranges from almost 20% in a depth of 150 nm to more than 1 μm. Both materials have a high oxygen content of about 50% on the surface. This value decreases at lower depth in the cast than in the FS state. The element Mg also displays a higher value close to the surface than further below, which is more pronounced in these measurements for the FS state. Other elements, mainly Ca, P, and S, originating from the lubricant, are found with up to 5 at-% directly at the surface.

**Figure 11:**
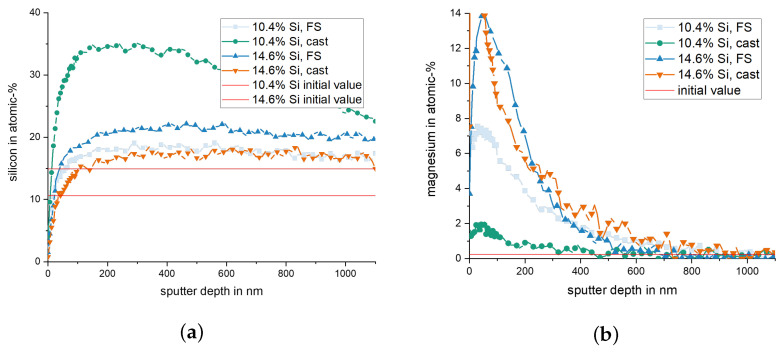
(**a**) Silicon content over sputtering depth (XPS) and the initial values for the different materials. (**b**) Magnesium content over sputtering depth.

The content in Si and Mg in the subsurface material of pins from 10.4% and 14.6% Si after wear testing is presented in Figure 11. A rise in the Si content is observed for all materials, up to a depth between 100 nm to 200 nm, remaining higher than the nominal alloying content up to a depth of > 1 μm. This effect is most pronounced for the samples with 10.4% Si, cast state, and 14.6% Si, FS state. For the element Mg, high values are measured directly in the surface, which tend to increase further within the first 100 nm below. At a depth of 400 nm (10.4% Si, cast) to 900 nm (10.4% Si, FS), the Mg values return to the alloys’ nominal content.

**Figure 12:**
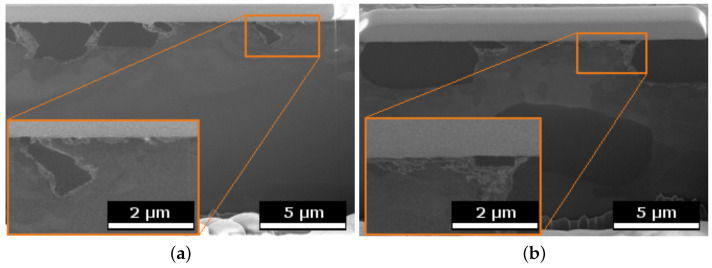
SEM images of FIB cross sections in worn surfaces of the pins with 10.4% Si; (**a**) as-cast, (**b**) FS.

Figure 12 shows SEM images of FIB cross sections of 10.4% Si pins, recorded underneath the worn surfaces. In the FS state, the Si phase particles (appearing dark in these images) have a rounder shape, and the material around them displays a thin, strongly deformed layer. The Si particles in the cast state have retained sharp edges, and a pronounced deformation of the surrounding material can be seen. In addition, the Al between the Si particles displays heavy deformation in the subsurface material, with a locally varying depth of up to ≈ 500 nm.

### 3.4. 42CrMo4 Discs

**Figure 13:**
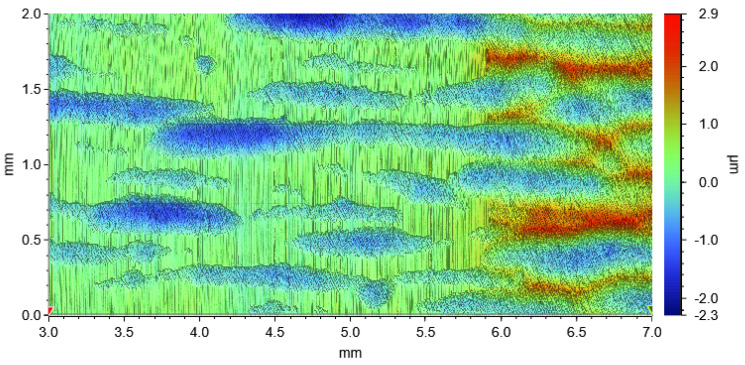
WLI image of disc with wear marks on the left, and original surface topography after finishing on the right.

Figure 13 shows a WLI image of the disc, displaying the worn surface on the left-hand side of the image and the original surface obtained after finishing on the right-hand side. A waviness and topography texture of the surface due to the finishing process when manufacturing the discs is clearly visible. Grooves in sliding direction (vertically in the image) are found only in those areas which protrude from the surface; the valleys in between seem not to have been affected by wear. The grooves are < 1 μm in depth. The difference in maximum height of the unworn areas to the height of the worn, grooved areas lies in a range of 2 μm to 3 μm.

**Figure 14:**
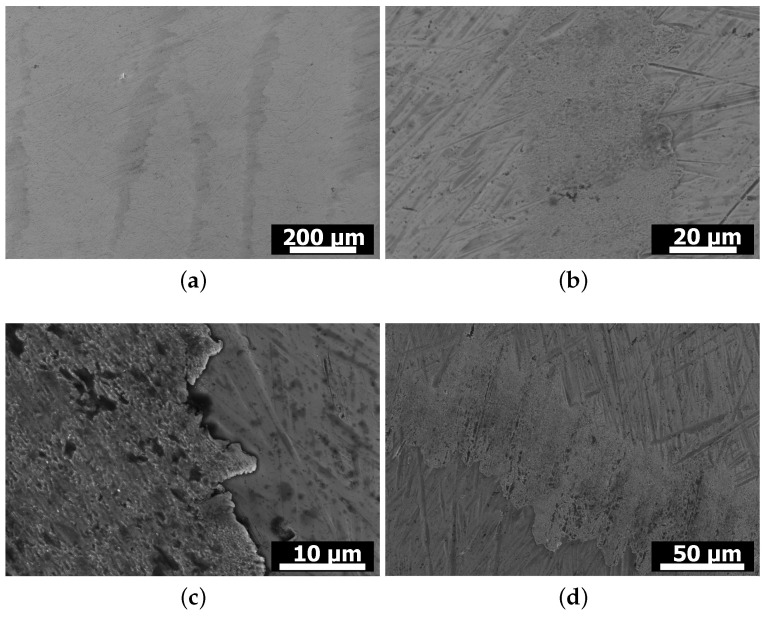
SEM images of worn disc surfaces after wear tests against (**a**–**c**) 10.4% Si FS pin and against (**d**) 6.6% Si FS pin.

In Figure 14, SEM images are displayed, showing details of the wear appearances on discs exposed to wear tests against a 10.4% Si FS pin (Figure 14a–c) and a 6.6% Si FS pin (Figure 14d). The grooves on the areas protruding from the surface can be clearly seen in Figure 14a–c show details of the worn, grooved areas. Here, it becomes visible that the grooves are not sharp edged but, rather, relatively smooth, as they are more difficult to observe at higher magnifications. The material in the disc surface appears to have been plastically deformed in the direction of relative sliding, leaving a smooth edge of the worn area where the pin counterface entered the contact, and a step in surface height on the edge where the pin left the contact, caused by disc material being deformed in sliding direction. The surface of the worn areas displays a fine roughness at high magnifications, as well as dark spots resulting from mechanochemical reactions of the lubricant and/or the atmosphere with the surface. The aforementioned wear appearances were found on all discs tested against all pin materials, as shown, e.g., by the image recorded on a disc after sliding against a 6.6% Si FS pin (Figure 14d), with a tendency to more pronounced wear and slightly larger worn, grooved areas for the 14.6% Si pins.

**Figure 15:**
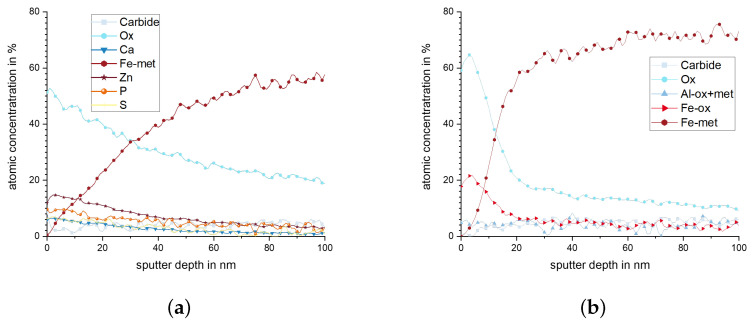
Chemical composition of elements with more than 4% atomic concentration (measured by XPS) of disk after wear test against a 10.4% Si FS pin (**a**) on worn area and (**b**) in valley.

In order to analyze the dark spots observed by SEM on the disc surfaces, XPS measurements were performed at two locations. Figure 15 shows the chemical compositions recorded by XPS at the disc on a worn, grooved area (Figure 15a) and in a valley displaying no signs of sliding contact (Figure 15b). Both measurements show a high amount of oxides in the surface and within the first nanometers of depth. In the unworn valley (Figure 15b), the concentration of metallic Fe rises sharply within the first 20 nm below the surface and chemical composition approaches the nominal values, although oxide phases are still detected up the maximum measured depth of 100 nm. This surface and subsurface state is assumed to be the result of the finishing process of the discs. In the worn area presented in (Figure 15a), the gradient in chemical composition and oxides content below the surface is significantly less steep, and, at 100 nm depth, the amount of oxide phase is still higher (Figure 14a). Additionally, zinc, phosphorus and sulfur are detected, which stem from the lubricant. Notably, although aluminum is detected in both areas, it is of very low amount (approximately 4 at-%). This reveals that no significant material transfer by adhesion from the pin surface has occurred.

## 4. Discussion

It is clearly observed that the weight loss caused by the conducted wear tests is strongly affected by the material processing condition for the two hypoeutectoid alloys (6.6% Si and 10.4% Si) but not for the hypereutectoid 14.6% Si alloy (Figure 7). For the 10.4% Si alloy, wear of pins in FS state is only 15% of the wear in the as-cast state. Still, weight loss of the hypereutectic alloy is lowest of all tested materials, both in as-cast and FS state, although the 10.4% Si alloy in FS state reaches nearly the same low wear. The friction curves (Figure 6) for the different materials show an unsteady behavior for the alloy with 14.6% Si, both in FS and cast state. The curves for 6.6% and 10.4% in FS state tend to be lower than for the 14.6% Si alloy, and the friction coefficient remains below 0.1 for all tests. The unsteady behavior, generally, may be related to the low contact pressures and the comparably soft Al alloys used as pins. It remains unclear exactly why the alloy with the lowest wear displays the highest fluctuations in coefficient of friction. A possible explanation might be the formation and loss of a third body or transfer film on the disk. Further experiments would be necessary to explain this phenomenon. A correlation between wear and coefficient of friction obviously does not exist. The main wear mechanisms are abrasion dominated by microploughing, combined with the breakout of the hard Si phase particles in all materials and material states, in spite of the observed differences in weight loss due to wear. For example, for the samples with 10.4% Si and 14.6% Si in FS state, similar weight loss was measured, although the worn surface of the latter material displays pronounced and wide grooves, while the first appears rather smooth (Figure 8). Obviously, the type of wear mechanisms acting does not show a simple correlation with the weight loss, either. It appears that similar mechanisms act in all materials but in different intensity, depending on the material state. Thus, while wear can be explained neither by the development of the friction coefficient nor by the general wear mechanisms, there is a clear correlation between the microstructure and wear. The eutectic point for Al-Si alloys lies at about 12.6% Si [34]. Therefore, the alloy with 14.6% Si is in contrast to the other two alloys investigated, a hypereutectic alloy. This leads to the formation of large bulky Si primary phase particles during solidification. In contrast, in hypoeutectic alloys the formation of Si primary phases in comparable quantity and size during solidification does not occur. During processing by FS, the needle-shape Si phase particles in all alloys are spheroidized, while the larger primary Si phase particles retain their shape and size, with only a slight rounding of the edges. It is clearly observable that those alloys which contain only the needle-shaped Si phase particles (i.e., 6.6% and 10.4% Si in as-cast state) display the highest wear. The needles fracture under the load and small fragments break out of the surface causing the observed high wear. After spheroidization by FS, the roundish Si phase particles display a higher resistance against breakouts and lower the resulting wear for 6.6% and 10.4% Si alloys. For the hypereutectic alloy with 14.6% Si, processing by FS shows no effect on the measured wear. Here, obviously, the needle-shaped Si phase particles in the as-cast state have no significant detrimental effect, since their spheroidization does not improve the wear resistance. It is noticeable that the globular Si phase particles in the alloy with 10.4% Si in FS state provide a comparable wear resistance as that of the alloy with 14.6% Si. Thus, the decisive factor for the resistance to wear is the combination of Si content and the shape and size in which the Si phases are present. The Al matrix surrounding the Si particles displays a dark, grainy appearance in the worn areas for all alloys, when studied from the surface (Figure 9). In the FIB cross sections of the surface of pins from 10.4% Si alloy (Figure 12), heavy deformation is observed in the Al solid solution below the surface and surrounding the Si phase particles. XPS measurements below the surface of the pins after the tests yield an increased Si content up to a depth of > 1 μm, with a maximum approximately 200 nm below the surface (compare Figure 11). High oxygen contents are observed up to 1000 nm below the surface and elements from the lubricant (Zn, Ca, P, S) are found to a depth of 100 nm to 200 nm below the surface of different pin materials. The increased Mg content of up to 14% close to the surface is particularly noticeable (Figure 10, Figure 11). These subsurface changes in the soft Al solid solution matrix may be caused by two main mechanisms: mechanical incorporation of wear particles and lubricant, and oxidation. It is visible from the FIB cross sections that Si particles detached from the surface and fractured under the tribological load are pressed into the deformed Al matrix. In the cast alloys, the needle-shaped Si grains fracture into a high number of small particles which are pressed into the surface more easily than the larger, globular particles, resulting in a pronounced Si accumulation in the subsurface material. The spherical Si phases in the FS material and the primary Si particles in the hypereutectic alloy break out of the surface less easily. Here, the hard Si particles are partially displaced by frictional shear forces, creating cavities, and causing detachment from the Al matrix behind and below the particles. This indicates that the Si particles are important for the derivation and distribution of the friction force and also possibly the contact pressure within the Al matrix. The deeper the Si particles extend into the material (i.e., the larger they are), the larger the area over which the force absorbed by the Si particle is distributed. Thus, the load on the Al matrix decreases with increasing size (depth) of the Si particles. This model applies to hypereutectic alloys in which Si is also present as a primary phase, as well as for the FS state of hypoeutectic alloys, in which the hard Si phases are present in spherical form. This is also the reason for the fact that the Si concentration in the XPS depth profile is the highest for the cast 10.4% Si alloy (see Figure 11). This sample showed higher wear than the other samples. The aluminum matrix is worn preferably, and the Si phase is enriched directly below the surface (see also FIB cross section Figure 12a). For the magnesium, we suggest a similar mechanism of enrichment as observed for the Si Phase. Magnesium is present in the material as Mg${}_{2}$Si. At the surface, oxidation also leads to the formation of MgO and SiO${}_{2}$, which can be recognized by a shift of the Si and Mg Peak in the XPS depth profile of the investigated samples [35]. However, Mg${}_{2}$Si phases, which are distributed in grains with diameters of several tenths to a few hundreds of nanometers, remain at the surface. The particles of Mg${}_{2}$Si are embedded in the surface, and the Al matrix is worn off disproportionately. The exact reason for the Mg enrichment would be subject of further work and could be elucidated by TEM, which is beyond the scope of the paper. The significantly harder steel also displays clear signs of surface alterations in the tribological contact for all Al alloy material states. While some mild grooves are visible, at high magnification, it becomes obvious that here mainly plastic flow occurred. This can be assumed to be due to ratcheting in the steel subsurface material during unidirectional sliding [36,37]. The analysis of chemical composition in the contact areas shows that oxides and elements from the lubricant are incorporated into the steel up to a depth of > 100 nm, correlating to the observed plastic interaction. Material transfer by adhesion from the Al pins was not found, ensuring the low coefficients of friction.

## 5. Conclusions

The changes in the microstructure induced by FS lead to an improvement in wear resistance for hypoeutectic alloys. The presence of Si phase particles in spherical form and of several μm in diameter is decisive for the wear resistance. Needle-shaped Si phase in the as-cast state fractures and small fragments detach from the surface easily. Larger spherical Si phase particles absorb the contact load and transmit it to the surrounding material. A distinction must be made between the effect of FS on hypoeutectic and hypereutectic alloys. With hypereutectic alloys, no changes in the wear rate between FS and cast material state were measured. The presence of needle-shaped Si phase particles in the as-cast state of the hypereutectic alloy showed no negative effect on wear resistance, as their spheroidization by FS did not yield any improvements. By changing the microstructure in hypoeutectic alloys, the wear rates could be improved by a factor of up to ≈ 6, so that the 10.4% Si alloy reaches the low wear rates of the hypereutectic alloy. The dominant wear mechanism on the Al alloy counterbodies is microploughing and the break out of Si phase particles. Si particles are embedded into the surrounding Al matrix surface, leading to a measurable enrichment in Si within the first 300 nm below the surface. In addition, lubricant and oxides formed on the surface during tribological loading are incorporated into the Al solid solution subsurface material. The steel discs display plastic deformation by ratcheting, as well as mechanical mixing with surface oxides and the lubricant under the mild wear conditions applied in this study.

## Figures and Tables

**Table 1:** Chemical composition of the custom-made Al alloys by element in weight-%.

alloy	Al	Si	Fe	Cu	Mn	Mg	Cr	Zn	Ti
6.6% Si	bal	6.61	0.198	0.016	0.009	0.253	0.002	0.003	0.017
10.4% Si	bal	10.37	0.201	0.011	0.009	0.245	0.002	0.003	0.014
14.6% Si	bal	14.6	0.227	0.011	0.010	0.232	0.002	0.001	0.013

**Table 2:** Hardness of pin materials.

Sample	HV0.2
6.6% Si Cast	86.7 ± 4.4
6.6% Si FS	84.3 ± 14.2
10.4% Si Cast	96.8 ± 6.2
10.4% Si FS	88.5 ± 3.5
14.6% Si Cast	103.2 ± 7.4
14.6% Si FS	114.6 ± 17.3

**Table 3:** Chemical composition of the disc material by element in weight-%.

Alloy	Fe	C	Si	Mn	Cr	Mo	S, P
42CrMo4	0.38–0.43	0.14–0.35	0.75–1.00	0.80–1.10	0.009	0.15–0.25	<0.035

## Data Availability

All data reported in this work can be requested from the corresponding author.

## Abbreviations

The following abbreviations are used in this manuscript:

FIB	Focused ion beam
FS	Friction surfacing
SEM	Scanning electron microscope
WLI	White light interferometry
XPS	X-ray photoelectron spectroscopy
